# Proteomic Analysis of Pathogenic Fungi Reveals Highly Expressed Conserved Cell Wall Proteins

**DOI:** 10.3390/jof2010006

**Published:** 2016-01-12

**Authors:** Jackson Champer, James I. Ito, Karl V. Clemons, David A. Stevens, Markus Kalkum

**Affiliations:** 1 Department of Molecular Immunology, Beckman Research Institute of City of Hope, Duarte, CA 91010, USA; jtchamper@yahoo.com; 2 Division of Infectious Diseases, City of Hope National Medical Center, Duarte, CA 91010, USA; JIto@coh.org; 3 California Institute for Medical Research, San Jose, CA 95128, USA; clemons@cimr.org (K.V.C.); stevens@stanford.edu (D.A.S.); 4 Division of Infectious Diseases and Geographic Medicine, Stanford University, Stanford, CA 94305, USA

**Keywords:** proteomics, vaccine candidates, *Aspergillus*, *Candida*, fungal pathogens

## Abstract

We are presenting a quantitative proteomics tally of the most commonly expressed conserved fungal proteins of the cytosol, the cell wall, and the secretome. It was our goal to identify fungi-typical proteins that do not share significant homology with human proteins. Such fungal proteins are of interest to the development of vaccines or drug targets. Protein samples were derived from 13 fungal species, cultured in rich or in minimal media; these included clinical isolates of *Aspergillus*, *Candida*, *Mucor*, *Cryptococcus*, and *Coccidioides* species. Proteomes were analyzed by quantitative MS^E^ (Mass Spectrometry—Elevated Collision Energy). Several thousand proteins were identified and quantified in total across all fractions and culture conditions. The 42 most abundant proteins identified in fungal cell walls or supernatants shared no to very little homology with human proteins. In contrast, all but five of the 50 most abundant cytosolic proteins had human homologs with sequence identity averaging 59%. Proteomic comparisons of the secreted or surface localized fungal proteins highlighted conserved homologs of the *Aspergillus fumigatus* proteins 1,3-β-glucanosyltransferases (Bgt1, Gel1-4), Crf1, Ecm33, EglC, and others. The fact that Crf1 and Gel1 were previously shown to be promising vaccine candidates, underlines the value of the proteomics data presented here.

## 1. Introduction

Pathogenic fungi, particularly *Aspergillus fumigatus* and *Candida albicans*, cause several thousand deaths per year among immunocompromised patients [1,2,3,4,5,6,7,8,9]. *Coccidioides* infection can occur in immunocompetent individuals [10]. Control and treatment of fungal infections is often challenging. Existing antifungal drugs are limited in effectiveness [11], and several species of fungi are becoming resistant to these treatments [12,13,14,15]. Thus, improved methods should be developed for the treatment and prevention of fungal infection.

Protein vaccines have been successful in several models of invasive fungal infection. For example, we have shown that vaccination with recombinant *A. fumigatus* Asp f3 (Pmp20) protected mice from aspergillosis following neutropenia or corticosteroid induced immunosuppression [16,17,18,19]. Additionally, vaccine formulations with Crf1 [20,21,22], Gel1 [21], and Pep2 [21] provided protection against aspergillosis in comparable experiments. Pmp1 from *Coccidioides* was an effective vaccine in a murine model of coccidioidomycosis [23], as were the proteins Pep1 [24] and Gel1 [25], Protection from candidemia has been conferred by immunization with recombinant Mdh1 [26], Sap2 [27], and Als3 [28,29,30], the last two have been investigated in clinical trials [31,32].

Several lines of evidence support the notion that developing a pan-fungal vaccine or at least a broad-spectrum vaccine that would protect against multiple fungal species may be feasible [33,34]. An ideal vaccine would protect against infection by multiple species of fungi by containing conserved epitopes that elicit both T cell and antibody responses. If protein based, such a vaccine candidate should be abundantly expressed as homologs by multiple species of fungi, preferably have cell wall localization, and be most dissimilar to any human protein. However, no large comparative proteomic studies have been published to date. Here, we report the quantitative proteomic analysis of 13 species of medically relevant fungi using a label-free MS^E^ (Mass Spectrometry—Elevated Collision Energy) approach [35,36,37,38,39]. These results serve as a beginning step in our efforts toward the development of a pan-fungal vaccine.

## 2. Materials and Methods

### 2.1. Fungal Strains

All *Aspergillus* and *Candida* strains were isolated from patients at the City of Hope National Medical Center under the institutional review board (IRB)-approved protocol #05024. These clinical isolates included *A. fumigatus* COH1 [16,19,40], *A. fumigatus* 685, *A. flavus* 654, *A. terreus* 638, *A. niger* 663, *A. nidulans* 730, *C. albicans* 671, *C. tropicalis* 708, *C. parapsilosis* 719, and *C. glabrata* 612 (numbers are referring to our internal collection of isolates). Identification was performed by the clinical laboratory at City of Hope and validated in mass spectrometry proteomics experiments by comparing the number of hits obtained from proteomic databases. Additional fungal strains included *Mucor* *circinelloides* strain NRRL 3631 (The U.S. Department of Agriculture, Agricultural Research Service Culture Collection), *Cryptococcus neoformans* var. *grubii* H99 (clinical isolate, Fungal Genetics Stock Center), *C. posadasii* strain Silveira (ATCC 28868) [41], and *S. cerevisiae* strain W303 (gift from Michael P. Rout, Rockefeller University, NY, USA). *S. cerevisiae* (“baker’s yeast”) was included because of the protective effect a heat-killed yeast antifungal vaccine has demonstrated previously [33,42,43,44], and because of growing numbers of reports of invasive *Saccharomyces* infections [45].

### 2.2. Culture Conditions

Both minimal and rich media were used in parallel for the culturing fungal organisms at 37 °C. These were the Czapek Dox (CD) minimal medium and the Potato Dextrose (CD) rich medium (both from Difco, Detroit, MI, USA). Although PD medium is typically used for the culturing of filamentous fungi, we used the same medium for yeasts to facilitate better proteomics comparisons between the genera. The inoculum size was 10^7^ spores or yeast cells for the molds *Aspergillus* and *Mucor*, or the yeasts *Candida*, *Cryptococcus*, and *Saccharomyces*, respectively. Cultures were harvested in the late exponential growth phase, 16–24 h after inoculation for PD medium and 2–5 days for CD medium, depending on the species. For *C. albicans*, it was necessary to supplement CD medium with 1 mg/mL casamino acids (Sigma Chemical Co., St. Louis, MO, USA) to ensure optimal growth (designated as CD+ medium). *C. posadasii* could not be cultured well in the media listed above and was therefore grown in a rich broth of 2% glucose and 1% yeast extract for 5 days at ambient temperature on a gyratory shaker at 150 rpm. Culturing and sample preparation of the hyphal form of *C. posadasii* was done in a BSL3 facility at the California Institute for Medical Research.

### 2.3. Sample Preparation

To prepare cell extracts (CE), yeasts were pelleted by centrifugation while hyphae of molds, except *C. posadasii*, were collected on filter paper and then partially dried using paper towels. Such yeast pellets and hyphal mats (~10 g, wet weight) were frozen under liquid nitrogen, and 50 µL of 100 mM PMSF and 50 µL of mammalian protease inhibitor cocktail (Sigma, St. Louis, MO, USA) was added. To disrupt cells, fungi were then ground in frozen state with a MM301 ball mill (Retsch, Haan, Germany) essentially as previously reported [46]. Briefly, frozen fungus samples were ground for three cycles at 30 Hz for 3 min while the temperature was maintained by submerging the stainless steel grinding jars into liquid nitrogen between cycles. Subsequently, the ground samples were suspended in Milli-Q purified deionized water (Millipore, Billerica, MA, USA) and sonicated with a Sonicator 3000 (Misonix, Farmingdale, NY, USA) using 60 × 0.75 s pulses at power setting 5 with 0.25-s intervals between pulses. The completeness of cell disruption was verified by microscopy. *C. posadasii* CE was prepared by vortex mixing of hyphae with 0.45 mm glass beads in 15 s cycles alternating with chilling in an ice bath. Homogenates were clarified by centrifugation and supernatants filtered through PVDF membranes with 0.22 µm pores (Millipore, Billerica, MA, USA). Culture filtrate (CF) was obtained by filtering spent fungal growth medium through membrane filters with 0.22 µm pores, addition of 10 µL fungal protease inhibitor cocktail (Sigma), and concentration by partial lyophilization to approximately 1 mL volume, followed by addition of trichloroacetic acid (TCA) to a final concentration of 200 mg/mL on ice. Samples were centrifuged at 4 °C, and the protein precipitates were retained. Cell wall (CW) extract was obtained by digestion of live fungal organisms for 8 hours at 30 °C in a digestion cocktail containing 1 mL with addition 10 µL fungal protease inhibitor cocktail (Sigma), 200 U/mL lyticase (Sigma), and 20 U/mL chitinase (Sigma), followed by centrifugation and filtration of the supernatant through a Millex PVDF filter with 0.22 µm pores (Millipore, Billerica, MA, USA).

### 2.4. Firefly Luciferase Quantification Standard for MS^E^

Recombinant Firefly Luciferase (FFL) was used to explore the dynamic range of MS^E^ quantification and as a standard. FFL cDNA was copied from the pGAL-FFL[SEL] plasmid (Addgene plasmid #1102 [47]), cloned into a *Nco*I/*Xho*I restricted pET28 vector (Novagen, EMD Biosciences, Madison, WI, USA) and expressed in BL21 *Escherichia coli*. The recombinant protein was purified via chromatography through a nickel nitrilotriacetic acid column and by gel filtration. Purity and identity was verified by SDS gel electrophoresis and mass spectrometry. FFL with >~98% purity was added to each sample at 500 fmol per μg protein before trichloroacetic acid precipitation as a quantification standard.

### 2.5. Mass Spectrometry

Proteins from CE and CW fractions were precipitated in 200 mg/mL TCA as described above. Approximately 12.5 μg of protein from each sample was suspended in a buffer containing 50% trifluoroethanol and 100 mM ammonium bicarbonate buffer at pH 8. Samples were reduced with tris(2-carboxyethyl)phosphine and alkylated with iodoacetamide. Additional ammonium bicarbonate buffer was added to reduce the trifluoroethanol concentration to 5%, and samples digested for 16 h at pH 8 with 6.25 μg/mL sequencing grade modified trypsin (Promega, Madison, WI, USA). MS^E^ mass spectrometric analysis was conducted using a SYNAPT G2 HD quadrupole time-of-flight (Q-TOF) mass spectrometer equipped with ion mobility separation unit and a nano Acquity 2D UHPLC system with a tile based Trizaic ion source (Waters, Milford, MA, USA). The mass range was 50–2000 Da, ion mobility separation (IMS) wave velocity was 650 m/s, and IMS wave height was 40 V. Solvents were 0.1% formic acid for buffer A and 0.1% formic acid in acetonitrile for buffer B. UHPLC chromatography was performed with a Trizaic for proteomics nanoTile containing 1.8 µm C18 particles in a 85 µm internal diameter × 100 mm column, by applying a linear solvent gradient over 50 min of 3% buffer B at the start, transitioning to 35% by minute 30, 50% by minute 34, 90% by minute 35, holding 90% through minute 38, transitioning to 3% buffer B by minute 43, and holding at 3% through minute 50. Collision energy was ramped automatically from 15–40 eV. Culturing, fractionation, and protein extraction were gradually improved over several trials for each fungus. The samples with the highest protein recovery and sequence coverages were then chosen for triplicate MS^E^ analyses on the mass spectrometer for protein quantification.

### 2.6. Protein Analysis

Data were analyzed using IdentityE (version 2.135.2.0, Waters, Milford, MA, USA) with default parameters in Protein Lynx Global Server (PLGS) (version 3.0.2, Waters, Milford, MA, USA). Search parameters included trypsin-derived peptides with up to one missed cleavage. Fixed modifications included carbamidomethyl addition to cysteine, and variable modifications included oxidized methionine. Mass tolerance was 0.025 Da for low energy ions and 0.01 Da for high energy ions. Protein databases searched included NCBI June 2014 versions of *A. fumigatus* Af293, *A. flavus* NRRL3357, *A. terreus* NIH2624, *A. niger* CBS 513.88, *A. nidulans* FGSC A4, *C. albicans* SC5314, *C. tropicalis* MYA-3404, *C. glabrata* CBS 138, *C. parapsilosis* CDC317, *C. posadasii* strain Silveira, *C. neoformans* var. *grubii* H99, and *S. cerevisiae* S288c. For identical proteins within each database, accession numbers were used with Swiss Protein as first priority, RefSeq as second priority, and others as third priority. Additionally, version 2.0 of *Mucor* *circinelloides* from the Joint Genome Institute was used. Common contaminant proteins were added to each database (Supplemental FASTA File 1). Scaffold 4.4.1 (Proteome Software, Portland, OR, USA) was used for quantification of proteins using a maximum false discovery rate of 5% as calculated by hits on the corresponding sequence-reversed database, with protein and peptide confidence levels adjusted to provide the maximum number of hits for the false discovery rate. MS^E^ protein quantifications were based on the average peak intensity of the top three peptides from each protein (or two for proteins with only two peptides available) and were normalized to femtogram of protein detected per microgram of total protein injected for each sample LC/MS^E^ analysis. For cases of peptides matching to multiple protein families, Scaffold assigned individual proteins using unique peptides and parsimony rules. Protein localization was predicted with the WoLF PSORT tool [48]. Matching of homologous proteins between fungal species was facilitated with a set of python scripts that included the following libraries: biopython, numpy, and matplotlib. The first script generated BLAST [49] homology data for each *A. fumigatus* protein in each of the fungal species examined by executing NCBI’s standalone BLASTP software version 2.2.30 with the default BLOSUM62 matrix, an expectation value of *e* < 0.05, a gap open value of 11, and a gap extension value of 1. With a second script, each MS^E^ quantified fungal protein homolog was identified in the BLASTP XML output using an expectation value of *e* < 10^−10^ and >30% sequence identity defined as the ratio of the number of identical residues over the length of the query sequence. Homologies to human proteins (Human RefSeq, NCBI Reference Sequence Database) were recorded for BLASTP expectation values of *e* < 10^−20^, due to the larger database size, and with >20% sequence identity. Finally, a third python script was used to generate heatmaps that displayed the amount of the closest quantified fungal protein homolog by color coding and its numerical value for percent sequence identity with the corresponding *A. fumigatus* protein. We used the GI (GenInfo Identifier) accession numbers to match all fungal protein homologs except for *Mucor circinelloides*. The latter was performed using the IGI accession numbers from the Joint Genome Institute database.

## 3. Results

### 3.1. MS^E^ Label-Free Protein Quantification

We assessed the dynamic range and linearity of the MS^E^ instrument response with a series of *A. fumigatus* CE samples that contained varying concentrations of spiked-in recombinant FFL. The instrument response was approximately linear at lower FFL concentrations from 135 to approximately 1400 fmol FFL per microgram of total protein (Figure S1), which corresponds to the concentration range in which nearly all of the fractionated fungal proteins were detected. For recombinant FFL, a saturation effect was observed outside the relevant range, at FFL concentrations >1400 fmol/µg total protein (Figure S1).

### 3.2. Mass Spectrometry of Fungal Fractions

**Figure 1:**
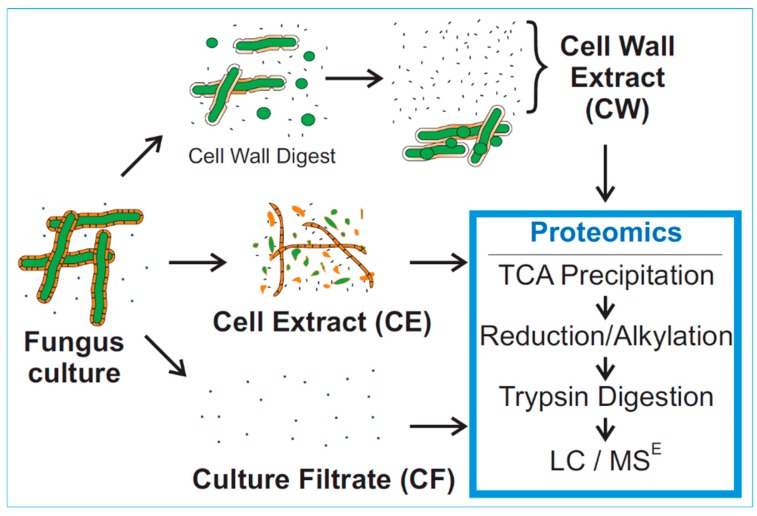
Workflow for generation of fungal protein fractions and processing for mass spectrometric analysis.

The workflow for the fractionation of fungal organisms to obtain proteins from whole cell extract (CE), culture filtrate (CF), and cell walls (CW) is shown in Figure 1. Proteins were identified and quantified separately for minimal and rich medium culture conditions. Detailed protein quantification results are listed in Tables S1–S18. These include data from two clinical isolates of *Aspergillus fumigatus* AFCOH1 and #685, *Aspergillus flavus*, *Aspergillus terreus*, *Aspergillus niger*, *Aspergillus nidulans*, *Candida albicans*, *Candida tropicalis*, *Candida parapsilosis*, *Candida glabrata*, *Cryptococcus neoformans* var. *grubii*, *Mucor circinelloides*, and *Saccharomyces cerevisiae* grown in PD medium, *A. fumigatus*, *A. flavus*, *A. terreus*, and *C. albicans* grown in CD medium, and in *Coccidioides posadasii* glucose yeast extract.

**Figure 2:**
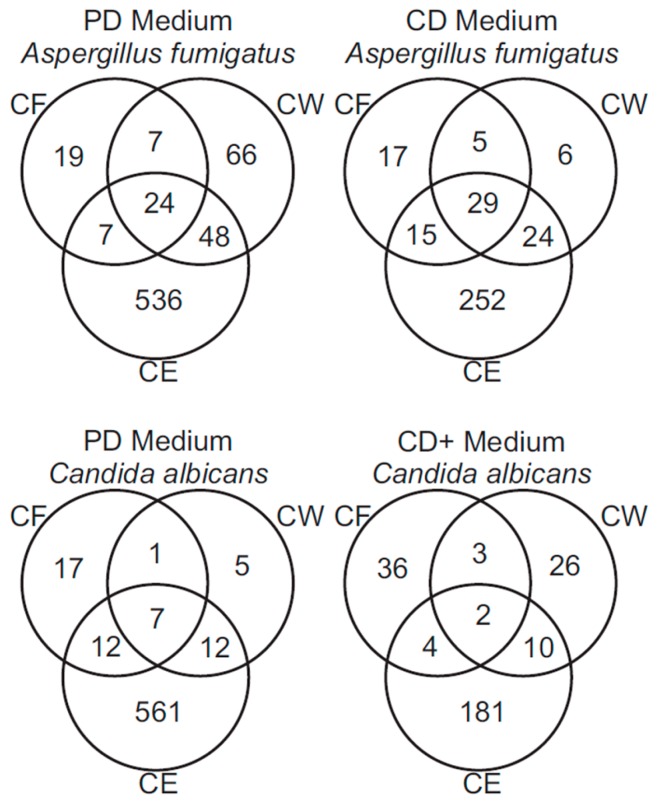
Number of proteins quantified in *A. fumigatus* and *C. albicans* fungal fractions. PD—Potato Dextrose Medium; CD—Czapek Dox Medium (+—with casamino acid supplementation); CE—Cell Extract Fraction; CF—Culture Filtrate Fraction; CW—Cell Wall Fraction. Protein numbers in overlapping sections of the Venn diagrams are of those shared by multiple fractions.

In total, several thousand unique proteins were identified and quantified by MS^E^ across all fractions (Tables S1–S18), ranging from 167 proteins for *A. flavus* cultured in CD medium to 707 for *A. fumigatus* grown in PD medium (Figure 2). Proteins (363–664) were identified in CE fractions from each species of fungus grown in PD medium, and 132–320 proteins were identified in CE fractions from each species of fungus grown in CD medium. CF fractions contained 15–131 identified proteins and CW fractions contained 17–194 identified proteins. Most proteins found in CD fractions were also found in PD fractions from the same species.

WoLF PSORT [48] predicted Surface and Secreted Localization (SSL) for proteins in the CF and CW fractions (Table 1). SSL proteins had either predicted extracellular (including cell wall and secreted) or membrane localization. The CF fractions contained, in total, 65% identified proteins possessing predicted SSL, with most individual fractions having higher levels of predicted SSL proteins. However, some CF fractions suffered more heavily from intracellular protein contamination, particularly *A. nidulans* with 43% SSL, *C. albicans* grown in CD+ media with 42% SSL, *C. parapsilosis* with 40% SSL, and *C. posadasii* with 34% SSL. The level of SSL in CW fractions was more varied, ranging from 27% in *C. parapsilosis* to 88% in *A. niger*, averaging 43% for proteins identified in all fungal species.

**Table 1:** Number of proteins quantified in each fungal fraction *.

Species	Growth Medium	CE	CF	%SSL	CW	%SSL
*Aspergillus flavus*	PD	508	25	80%	86	38%
*Aspergillus flavus*	CD	132	41	76%	36	72%
*Aspergillus fumigatus*	PD	615	57	70%	145	38%
*Aspergillus fumigatus* 685	PD	510	62	95%	194	33%
*Aspergillus fumigatus*	CD	320	66	77%	64	70%
*Aspergillus nidulans*	PD	450	68	43%	46	61%
*Aspergillus niger*	PD	511	35	97%	17	88%
*Aspergillus terreus*	PD	580	25	96%	84	50%
*Aspergillus terreus*	CD	204	27	56%	45	64%
*Candida albicans*	PD	592	38	61%	25	60%
*Candida albicans*	CD+	197	45	42%	41	37%
*Candida glabrata*	PD	363	33	91%	57	33%
*Candida parapsilosis*	PD	664	48	40%	78	27%
*Candida tropicalis*	PD	375	24	63%	51	41%
*Coccidioides posadasii*	PD	403	131	34%	53	28%
*Cryptococcus neoformans*	PD	609	15	80%	30	40%
*Mucor circinelloides*	PD	419	25	88%	40	53%
*Saccharomyces cerevisiae*	PD	481	16	94%	46	28%

* PD—Potato Dextrose Medium; CD—Czapek Dox Medium (+—with casamino acid supplementation); CE—Cell Extract Fraction; CF—Culture Filtrate Fraction; CW—Cell Wall Fraction; SSL—Surface or Secreted Localization.

### 3.3. Analysis of A. fumigatus and C. albicans Proteins

Proteins from the major opportunistic pathogens *A. fumigatus* and *C. albicans* were assessed for fraction overlap. In total, 707 identified proteins were quantified in fractions from *A. fumigatus* grown in PD medium, 348 in fractions from *A. fumigatus* grown in CD medium, 615 proteins in fractions from *C. albicans* grown in PD medium, and 262 in fractions from *C. albicans* grown in CD+ medium (Figure 2). CF and CW fractions usually had considerable overlap between identified proteins in *A. fumigatus*, with less overlap in *C. albicans*, particularly when grown in CD+ medium. However, each fraction also had significant numbers of proteins not found in other fractions, several of which were abundant.

### 3.4. Interspecies Protein Comparisons

To determine the proteins that are conserved between the different fungal species, the proteomics results for each fungal fraction and culture condition were compared to each other. To avoid ambiguities, interspecies comparisons were based on the protein sequence of each identified protein, and not on protein names or accession numbers. NCBI’s BLASTP revealed many *A. fumigatus* and *C. albicans* homologs of abundantly expressed proteins in CE fractions and SSL proteins in CF and CW fractions (Tables S1–S18). These homologs were compared to proteins identified in *A. fumigatus*. Within these Supplemental Tables, the percent sequence identity is given.

Abundantly expressed *A. fumigatus* proteins usually had high degrees of homology with other *Aspergillus* species. For *A. flavus*, *A. terreus*, *A. niger*, and *A. nidulans*, homologies to *A. fumigatus* proteins ranged from 84%–87% for CE and 53%–64% SSL proteins (Table 2). Homology levels were nearly as high when comparing *A. fumigatus* and *C. posadasii* proteins, averaging 79% for CE and 49% for SSL proteins. *A. fumigatus* protein homology levels with other species including *M. circinelloides*, *Candida* species, *C. neoformans* var. *grubii*, and *S. cerevisiae*, were approximately 10%–25% lower than for the *Aspergilli* and *C. posadasii* (Table 2).

*C. albicans* proteins possessed high degrees of homology to proteins from *C. tropicalis* and *C. parapsilosis*, moderate homology to *C. glabrata* and *S. cerevisiae*, and lower levels of homology with other species (Table 3).

Considering all of the interspecies protein homology comparisons observed, it became clear that SSL proteins had lower levels of interspecies similarity than the intracellular proteins. However, SSL protein homologies were still substantial with 33%–64% sequence identity.

**Figure 3:**
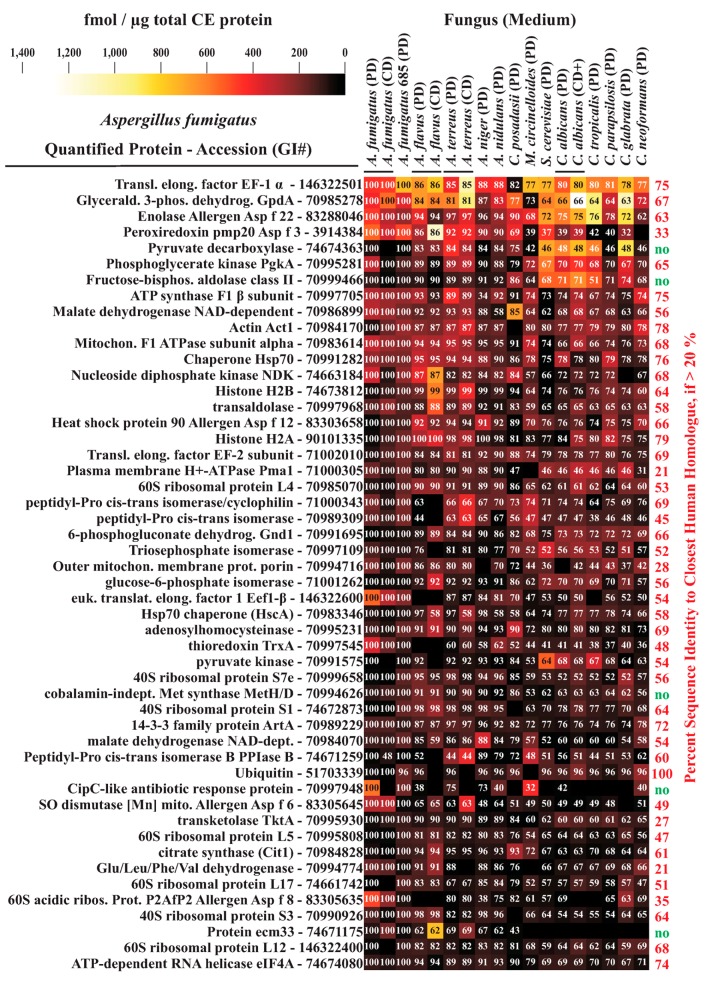
Quantities and homologies of proteins from fungal cell extracts. The color gradients in the heat map represent quantities of the closest detected homolog, and the numbers in each box give the degree of homology as percent sequence identity to the corresponding protein homolog in *A. fumigatus*. Black boxes that contain no numbers indicate absence of a detected homolog.

**Figure 4:**
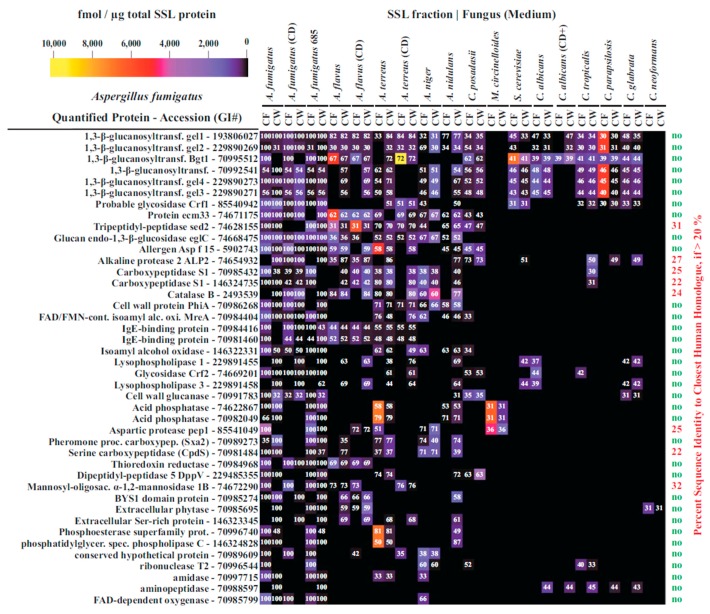
Quantities and homologies of fungal proteins with secreted or surface localization (SSL). The color gradients in the heat map represent quantities of the closest detected homolog in culture filtrate (CF) or cell wall (CW) extracts. The numbers in each box give the degree of homology as percent sequence identity to the corresponding protein homolog in *A. fumigatus*. Black boxes that contain no numbers indicate absence of a detected homolog.

The 50 proteins identified as most abundant in CE fractions in all species analyzed are displayed in a heat map using color coding to visualize protein amounts and overlaid numbers to indicate the level of sequence identity respective to the homologous *A. fumigatus* protein (Figure 3). Forty-five of these fungal proteins had significant homology (>20%) to human proteins with an average fungus-to-human sequence identity of 59%. One protein, *A. fumigatus* ubiquitin had 100% sequence identity to human ubiquitin and slightly less homology to other fungal ubiquitins. The vaccine candidate Pmp20 (Asp f3) had highly expressed homologs in all species analyzed except *C. neoformans* var. *grubii* and it also had only 33% identity to its human homolog. The 42 *A. fumigatus* SSL proteins with the greatest number of highly expressed homologs among the species analyzed are displayed in a similar heat map in Figure 4. Of the 42 SSL proteins, only 8 had some homology (>20%) to human proteins. Those were Sed2, Alp2, two carboxypeptidase S1 proteins, catalase B, Pep1, CpdS, and a mannosidase. However, the latter averaged only 26% sequence identity when comparing the respective *A. fumigatus* protein to its closest human homolog.

**Table 2:** Homology comparisons for the 50 most abundant CE proteins and all SSL proteins of *Aspergillus fumigatus* *.

Fraction	*A. flavus*	*A. flavus* CD	*A. terreus*	*A. terreus* CD	*A. niger*	*A. nidulans*	*C. posadasii*	*M. circinelloides*	*S. cerevisiae*	*C. albicans*	*C. albicans* CD+	*C. tropicalis*	*C. parapsilosi*	*C. glabrata*	*C. neoformans*
# Proteins, CE	47	40	50	47	49	50	46	46	48	48	47	46	48	46	48
Average % Identity	85 ± 14	87 ± 10	85 ± 12	84 ± 12	85 ± 15	84 ± 13	79 ± 13	62 ± 12	64 ± 12	66 ± 12	65 ± 12	64 ± 13	66 ± 13	65 ± 13	63 ± 12
# Proteins, CF	13	17	25	12	25	11	17	3	7	9	1	9	7	10	1
Average % Identity	56 ± 19	53 ± 17	60 ± 15	60 ± 17	56 ± 14	57 ± 12	49 ± 11	33 ± 3	42 ± 5	42 ± 5	39 ± 0	40 ± 6	37 ± 7	42 ± 6	31 ± 0
# Proteins, CW	25	23	34	25	17	30	13	3	9	7	4	12	9	12	1
Average % Identity	59 ± 17	62 ± 16	61 ± 16	64 ± 15	53 ± 16	59 ± 14	50 ± 12	33 ± 3	42 ± 6	41 ± 6	41 ± 7	39 ± 8	39 ± 7	41 ± 5	31 ± 0

* #—number of; PD—Potato Dextrose Medium; CD—Czapek Dox Medium (+—with casamino acid supplementation); CE—Cell Extract Fraction; CF—Culture Filtrate Fraction; CW—Cell Wall Fraction. ± indicates standard deviation.

**Table 3:** Homology comparisons for the 100 most abundant CE proteins and all SSL proteins of *Candida albicans* *.

Fraction	*A. fumigatus*	*A. fumigatus* CD	*A. fumigatus* 685	*A. flavus*	*A. flavus* CD	*A. terreus*	*A. terreus* CD	*A. niger*	*A. nidulans*	*C. posadasii*	*M. circinelloides*	*S. cerevisiae*	*C. tropicalis*	*C. parapsilosi*	*C. glabrata*	*C. neoformans*
# Proteins, CE	88	74	95	93	75	97	91	91	95	83	93	96	97	98	99	96
Average % Identity	66 ± 11	65 ± 13	66 ± 12	66 ± 13	66 ± 14	65 ± 13	64 ± 14	67 ± 11	65 ± 13	65 ± 13	63 ± 13	74 ± 12	90 ± 10	87 ± 10	74 ± 12	63 ± 12
# Proteins, CF	5	5	5	3	2	3	2	4	3	7	1	7	15	14	18	0
Average % Identity	41 ± 5	41 ± 5	41 ± 5	40 ± 4	40 ± 5	41 ± 6	42 ± 2	39 ± 5	37 ± 6	41 ± 9	32 ± 0	50 ± 7	56 ± 16	54 ± 16	47 ± 10	0 ± 0
# Proteins, CW	10	7	9	8	7	7	6	4	6	6	1	11	15	16	13	0
Average % Identity	38 ± 5	40 ± 5	39 ± 5	40 ± 5	40 ± 6	39 ± 5	37 ± 4	39 ± 5	41 ± 6	43 ± 9	32 ± 0	46 ± 8	60 ± 17	55 ± 16	44 ± 10	0 ± 0

* #—number of; PD—Potato Dextrose Medium; CD—Czapek Dox Medium; CE—Cell Extract Fraction; CF—Culture Filtrate Fraction; CW—Cell Wall Fraction. ± indicates standard deviation.

Among the most abundantly expressed proteins only a few significant differences were discovered when comparing fungi cultured in rich *versus* minimal medium. CE fractions from fungi grown in the rich PD medium contained many more identified proteins than CE fractions from fungi grown in minimal CD medium (363–615 proteins for PD medium *vs.* 132–320 for CD medium). It appears that the detection threshold was substantially lower in PD fractions (0.5–3 *versus* 5–15 fmol per microgram total protein), possibly because fractions from CD grown cultures contained more highly abundant proteins, which may have masked less abundant peptides.

### 3.5. Functional Annotation of A. fumigatus and C. albicans Proteins

**Figure 5:**
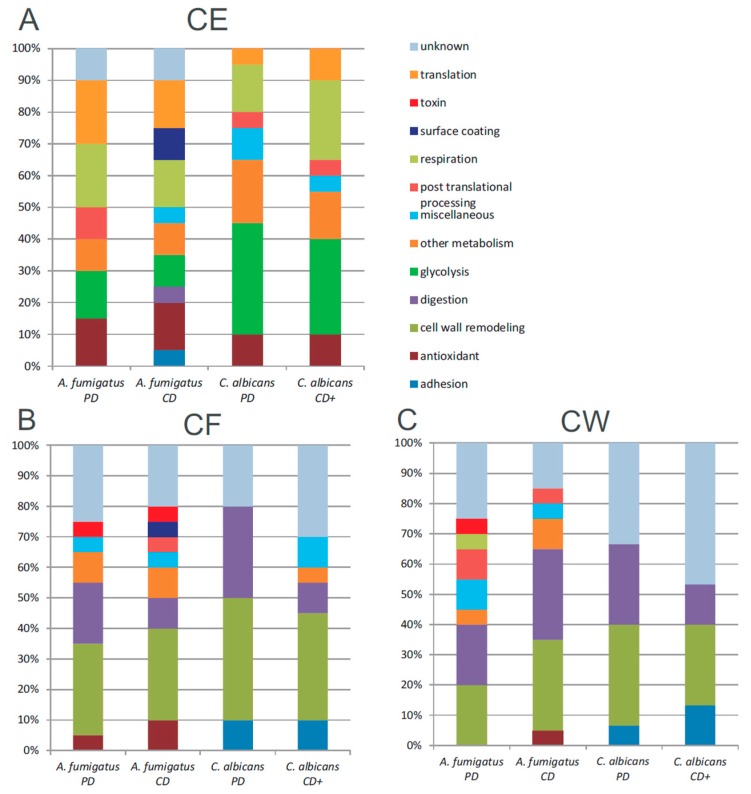
Functional annotation of the most abundant proteins in *A. fumigatus* and *C. albicans*. PD—Potato Dextrose Medium. CD—Czapek Dox Medium (+—with casamino acid supplementation). (**A**) CE—Cell Extract Fraction (SSL proteins); (**B**) CF—Culture Filtrate Fraction (SSL proteins); (**C**) CW—Cell Wall Fraction.

Functional annotation was performed manually for the 20 most abundant proteins in *A.* *fumigatus* and *C. albicans* fractions. For CF and CW fractions, only proteins with predicted SSL were considered, and only 15 SSL proteins were available for analysis in *C. albicans* CW fractions. In CE fractions, 11–15 proteins had known functions in glycolysis, respiration, translation, and as antioxidants (Figure 5A). *C. albicans* had more abundant proteins involved in glycolysis (6–7 of the 20 most abundant compared to 2–3 for *A. fumigatus*), while *A. fumigatus* had more abundant proteins devoted to translation (3–4 of the 20 most abundant compared to 1–2 for *C. albicans*). In CF fractions, 4–6 proteins of unknown function were detected, as were 2–6 proteins involved in digestion, usually proteases or amylases (Figure 5B). Interestingly, 6–8 proteins involved in cell wall remodeling were also detected in all fractions. *C. albicans* fractions contained more cell-cell adhesion proteins, while *A. fumigatus* fractions contained more proteins of miscellaneous function, including antioxidants and metabolism-related proteins. These same patterns were retained in CW fractions (Figure 5C).

## 4. Discussion

It was the goal of our study to identify abundantly expressed fungi-typical proteins that share only little homology to human proteins. Such proteins should be interesting to future studies that are aimed to develop vaccine candidates or drug targets. To refine the localization of such proteins, we applied a fractionation strategy that focused on cytosolic, cell wall, and secreted proteins. As common with cellular fractionation methods, none provides perfect enrichment. We carefully conducted enzymatic cell wall digestion and used mass spectrometry to guard for the eventual release of cytosolic proteins, which would have indicated over digestion. Bioinformatic analysis indicated that an average of 71% of CF proteins, and ~48% of CW proteins had predicted SSL (Table 1), supporting substantial enrichment. Naturally, CW and CF fractions also contained several proteins with predicted intracellular localization. Nevertheless, our fractionation method still produced acceptably low levels of cross contamination, and therefore each fraction represents a distinct proteome. Predicted intracellular proteins in CW and CF fractions may in fact be cell wall or secreted proteins with unknown export signals. In fact, formation of extracellular vesicles by fungi has been reported previously [50,51,52]. In addition, the detection of cytosolic proteins in CW and CF fractions can result from natural autolysis of cells, which may release intracellular proteins that adhere to extracellular cell surfaces. Thus, such proteins are likely to form a natural part of the cell surface and the secreted proteome of most fungi. Indeed, a previous study found several predicted intracellular proteins on the extracellular surface of *Cryptococcus*, which were then successfully tested as vaccine candidates [53]. Here, we limited our set of abundantly expressed extracellular or surface localized proteins based on predicted SSL signals. As discussed below, abundantly expressed SSL proteins are largely conserved between fungal species and most distant from any human homologs.

Functional annotation of the most abundant proteins in *A. fumigatus* and *C. albicans* CE fractions revealed similar functions for fungi grown in PD and CD media (Figure 5). In CE fractions, proteins with a variety of biochemical functions related to metabolism and protein synthesis were found, as expected. However, *A. fumigatus* appeared to have lower expression levels of proteins involved in glycolysis and higher expression of proteins involved in translation. This may be because yeast such as *C. albicans* may rely more on glycolysis for energy in non-limiting conditions, while filamentous fungi rely more on aerobic respiration, consistent with previous microarray studies [54]. In CF and CW fractions of *A. fumigatus* and *C. albicans*, many abundant proteins of unknown function were detected (Tables S1, S2, S13 and S14), which may be good targets for future functional investigations regarding the interaction of fungi with their environment. Interestingly, several proteins involved in cell wall remodeling were found not just in CW fractions, but in CF fractions as well. These proteins may have been shed during the course of cell wall remodeling, or perhaps also serve other roles in carbohydrate processing. Such proteins, such as Crf1 and EglC in *A. fumigatus*, may also serve to inhibit the growth of other fungi by degrading their cell walls. *C. albicans* CF and CW fractions contained adhesion proteins allowing for cell-cell interaction, and consistently were not found in *A. fumigatus* CF and CW fractions. *A. fumigatus*, on the other hand, contained several abundant protein types of varying function in the CW and CF fractions not found in *C. albicans*, including established virulence factors such as the serine protease Asp f15 and ribotoxin Asp f1 [55].

The cell wall of pathogenic fungi plays an important role in host-pathogen interaction. Other studies have increasingly focused on proteins from this cellular site [56], and our study provides a first look at the cell wall proteomes, as well as other fractions of several species (Table 4). The CW proteins identified from hyphae in our study have very little overlap with those of the conidial cell wall proteome of *A. fumigatus* identified in a different study [57]. Thus, it appears that the *A. fumigatus* cell wall proteome undergoes major changes between the conidia and hyphae stages of the fungal lifecycle, particularly when the fungi are grown in rich medium, which is consistent with previous reports [58]. The intracellular proteome of conidia is also different from that of hyphae, though to a lesser extent [59,60]. The proteins detected in our *A. fumigatus* CE, CF, and CW fractions were very similar in expression levels to our previous study in both PD and CD medium [61]. Our cell wall proteomes had few proteins in common with earlier studies on *C. albicans* [62,63] and *C. glabrata* [64], most likely due to differences in PD medium and the more commonly used yeast extract peptone dextrose medium. For example the Ecm33 homologs, which were abundant in the filamentous fungi studied here, were not detected in the fractions from yeast.

Our CF protein identifications had a fair amount of overlap with previous secretome studies of *A. fumigatus* [65,66], *A.* *terreus* [67], *A. niger* [68], *A. flavus* [69,70], *C. albicans* [71,72], and *S. cerevisiae* [73]. Differences between our results and these studies are likely due to differences in culture conditions, growth media, and sample preparation methods. Our protein identifications from CE fractions were also broadly similar to previous studies covering *A. fumigatus* [59,74], *A. flavus* [75,76], *A. nidulans* [77], *C. albicans* [78,79,80], *C. glabrata* [81], *C. posadasii* [23], *C. neoformans* [53,82], and *S. cerevisiae* [83,84,85,86]. We also identified many of the immunogenic proteins found in a study on *C. parapsilosis*, including an ATP synthase, an enolase, GAPDH, and others [87].

**Table 4:** Recently Published Fungal Proteomic Studies *.

Species	CE	CF	CW
*Aspergillus flavus*	[75,76]	[69,70]	-
*Aspergillus fumigatus*	[58,59,60,61,74]	[61,65,66]	[57,61]
*Aspergillus nidulans*	[77]	-	-
*Aspergillus niger*	-	[68]	-
*Aspergillus terreus*	-	[67]	-
*Candida albicans*	[78,79,80]	[71,72]	[62,63]
*Candida glabrata*	[81]	-	[64]
*Candida parapsilosis*	[87]	-	-
*Candida tropicalis*	-	-	-
*Coccidioides posadasii*	[23,61]	-	-
*Cryptococcus neoformans*	[53,82]	-	-
*Mucor circinelloides*	-	-	-
*Saccharomyces cerevisiae*	[83,84,85,86]	-	[73]

* Fraction: CE—Cell Extract, CF—Culture Filtrate, CW—Cell Wall; All fungi and fractions mentioned in the table were analyzed in this present study. Hyphens mark the absence of other published studies.

In recent studies, we tested serum from mice infected with *C. posadasii*, *C. albicans* or *Paracoccidioides brasiliensis* on a 4800 element ordered protein array of *Saccharomyces cerevisiae* to profile cross-reactive antigen proteins [88]. These sera detected 60 to 130 proteins each, including five that we had reported in our initial studies using immunoblotting and MS [61]; 16 detected proteins were common to all. Interestingly, no cell wall or GPI-anchored proteins were detected using this method of screening, possibly due to low titers of antibody in the sera or stimulation of primarily a cell-mediated immune response. This difficulty is obviated by the MS methodology, which allows us to analyze all proteins present in a sample preparation or cellular fraction, even if they do not stimulate a strong antibody response.

Conserved fungal proteins could become components of vaccines that protect against multiple species of fungi. For example, crude complex vaccines such as heat-killed *Saccharomyces cerevisiae* has been protective in experiment models of aspergillosis [43], candidiasis [89], coccidioidomycosis [42], cryptococcosis [90], and mucormycosis [44]. It is striking that the aspergillosis vaccine candidate Asp f3 from *A. fumigatus* [16,17,18,19] shares 70% sequence identity with the coccidioidomycosis vaccine candidate Pmp1 from *C. posadasii* [23]. Similarly, Pep2 from *A. fumigatus* [21] and Pep1 from *C. posadasii* [24] share 77% sequence identity, and Gel1 from *A. fumigatus* [21] and from *C. posadasii* [25] have 69% identity. Furthermore, the *A. fumigatus* protein Crf1 protects not only against aspergillosis, but also candidiasis [22], despite exhibiting only 48% identity across 66% of the homologous *C. albicans* protein. An ideal vaccine candidate would be most effective if it were expressed throughout the growth cycle of fungi, particularly by conidia in filamentous fungi. Additionally, such a protein vaccine candidate would be localized to the cell wall, to allow for easier immune access, including by antibodies. Finally, when selecting proteins as potential vaccine candidates, it is desirable for the protein to be expressed at a high level by the fungus during infection *in vivo*. Using studies *in vitro*, the chance of this can be maximized by using proteins that are expressed in multiple growth conditions. Such expression would likely also be conserved *in vivo*, as supported by other published observations [61,88]. A superior vaccine may be created by selecting immunogenic epitopes of several proteins and combining them into a recombinant protein. In support of this strategy, a combination of recombinant Pep1, Alm1, and Plb1 in a vaccine formulation provided increased protection against *Coccidioides* infection compared to each individual protein alone [91]. Indeed, the flexibility of this strategy may allow for a single multivalent protein vaccine to be designed for protection against different species of fungi using epitopes of multiple proteins from several species. If such epitopes were linked by cathepsin cut sites, immune processing in the endolysosome may be enhanced, leading to a more efficient vaccine. Efficiency could be further increased by selecting individual epitopes that provide protection against multiple species of fungi.

## 5. Conclusions

We have discovered several cell wall proteins with homology and expression across different species (Figure 4) that that may be suitable candidates for identification of these protective epitopes. Our best candidates with high expression in multiple species of fungi and no homology to human proteins are the 1,3-β-glucanosyltransferases, including Gel1-4, Bgt1, and their homologs. Other interesting candidates discovered are Crf1, Ecm33, and EglC. It is noteworthy that Crf1 and Gel1 have already been shown to be promising vaccine candidates, while the other proteins remain to be tested. Vaccine trials with our abundant homologous fungal candidate proteins (Figure 4) should be performed to assess protection against multiple species of fungi.

## Supplementary Materials

Supplementary File 1
