# Supplementary material for: Proteomic Analysis of Pathogenic Fungi Reveals Highly Expressed Conserved Cell Wall Proteins
Source: J Fungi (Basel). 2016 Jan 12;2(1):6. doi: 10.3390/jof2010006 (PMC4747415; doi:10.3390/jof2010006)
Supplement: Supplementary File 1 [file jof-02-00006-s001.zip › Suplementary Files/Supplemental Figure 1.docx]

Supplementary Materials: Proteomic Analysis
of Pathogenic Fungi Reveals Highly Expressed Conserved Cell Wall Proteins

Jackson Champer, James I. Ito, Karl V. Clemons, David A. Stevens and Markus Kalkum

**
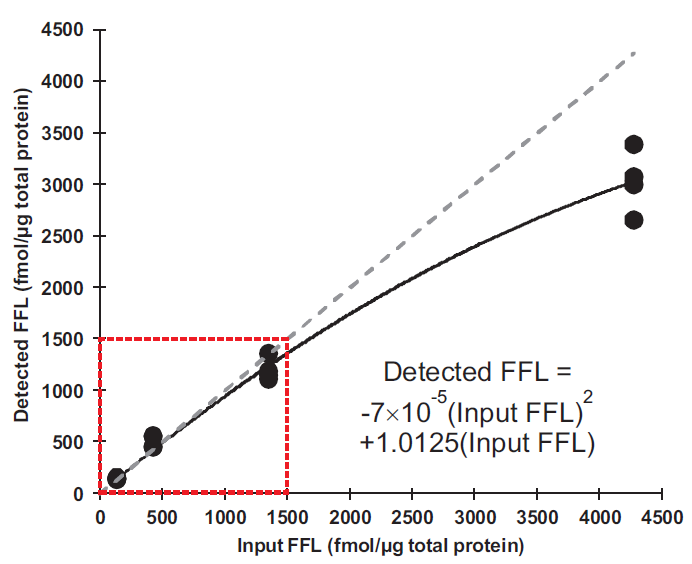
**

**Figure S1.** Dynamic range and linearity of MS^E^ (Mass Spectrometry—Elevated Collision Energy) for label-free protein quantification in the presence of *Aspergillus fumigatus* cell extract. The dashed red box indicates the range of most quantified proteins, and the grey dashed line shows an ideal 1:1 ratio. Data from 16 independent LC-MS^E^ analyses. FFL—recombinant Firefly Luciferase.
